# Supplementary material for: Influence of Seasonality and Public-Health Interventions on the COVID-19 Pandemic in Northern Europe
Source: J Clin Med. 2024 Jan 6;13(2):334. doi: 10.3390/jcm13020334 (PMC10816378; doi:10.3390/jcm13020334)
Supplement: Supplementary file 1 [file jcm-13-00334-s001.zip › jcm-2750738-Supplementary/jcm-2750738-Supplementary Figures with legends.pdf]

Supplementary data for

The influence of non-pharmaceutical interventions, vaccination and coronavirus seasonality on the progression of the COVID-19 pandemic in Northern Europe.

Gerry A Quinn, Michael Connolly, Norman E Fenton, Steven J Hatfill, Paul Hynds, Coilín ÓhAiseadha, Karol Sikora, Willie Soon and Ronan Connolly

Supplementary Tables

Table S1. Estimated time period in weeks between the peaks of deaths and cases during the 3 waves of the COVID-19 pandemic in Northern European countries. Deaths and cases both measured per 100,000. Data covering the period 1 March 2020 to 6 May 2023 was taken from "Our World in Data", https://ourworldindata.org/coronavirus; accessed 05/07/23**.**

Country	1st Wave	2nd Wave	3rd Wave
**Ireland**	1	3	3
**UK**	1	2	2
**Sweden**	1	2	4
**Denmark**	1	3	4
**Finland**	1	1	3 or 4
**Norway**	2	3	6 or 7

Supplementary Figures

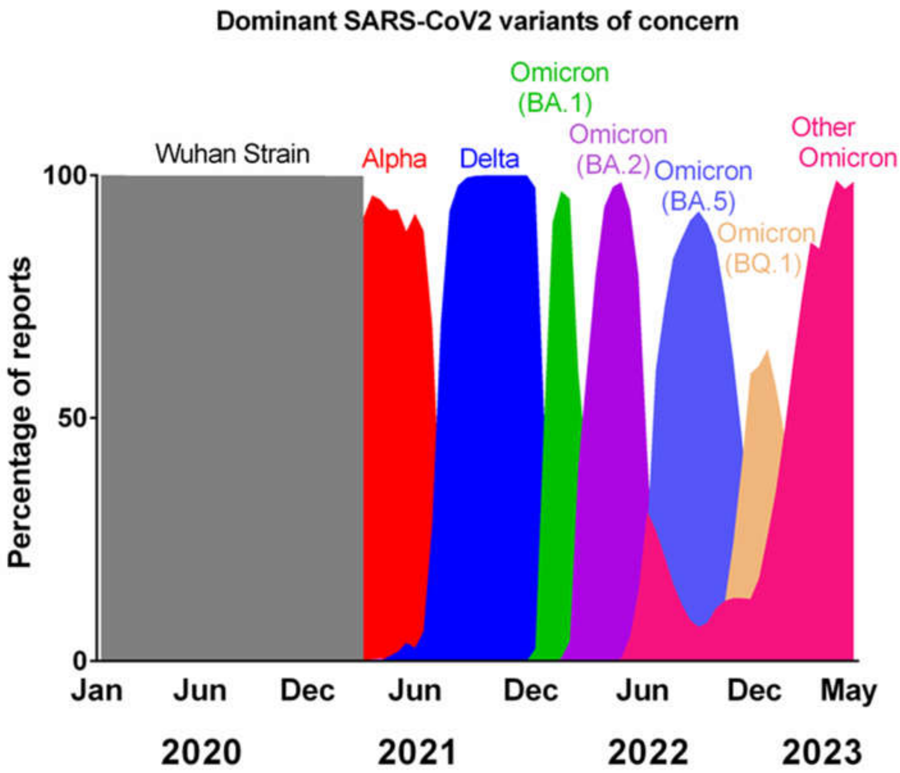

Figure S1. Development of the main SARS-CoV2 variants over the course of the COVID-19 pandemic. Data covering the period 1 March 2021 to 8 May 2023. Data sourced from GISAID, via CoVariants.org -accessed 1 November 2023 - Note: Recently-discovered or actively-monitored variants may be overrepresented, as suspected cases of these variants are likely to be sequenced preferentially or faster than other cases. Data from Our-WorldInData.org/coronavirus. More detailed breakdown of variants of concern can be found in Excel File S1

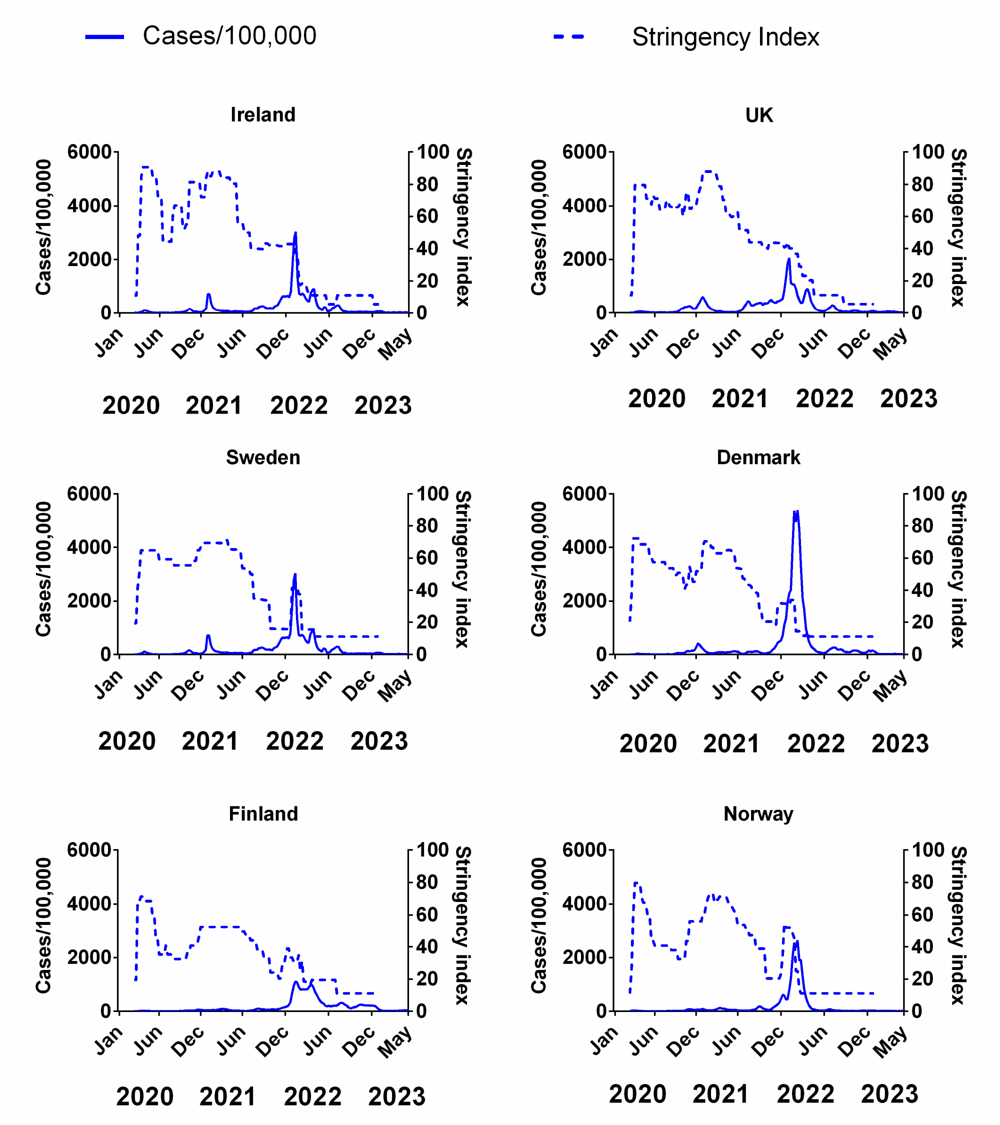

Figure S2a. Changes in NPI stringency compared to the progression of the COVID-19 pandemic in Northern Europe as measured by new cases. Cases per 100,000 of the population. Data covering the period 1 March 2020 to 6 May 2023 was taken from "Our World in Data", https://ourworldindata.org/coronavirus; accessed 05/07/23 [55].

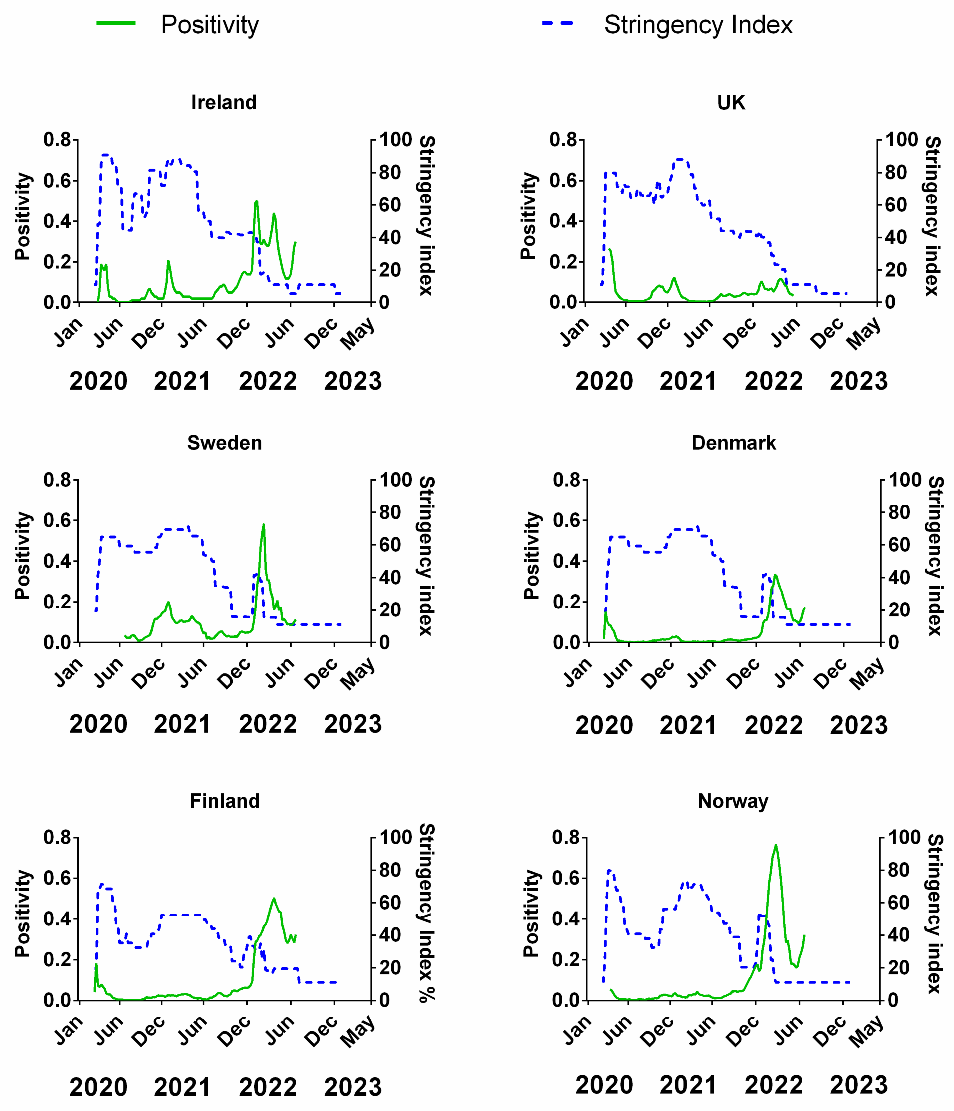

Figure S2b. Changes in NPI stringency compared to the progression of the COVID-19 pandemic in Northern Europe as measured by the positivity rate. Data covering the period 1 March 2020 to 6 May 2023 was taken from "Our World in Data", https://ourworldindata.org/coronavirus; accessed 05/07/23 [55].

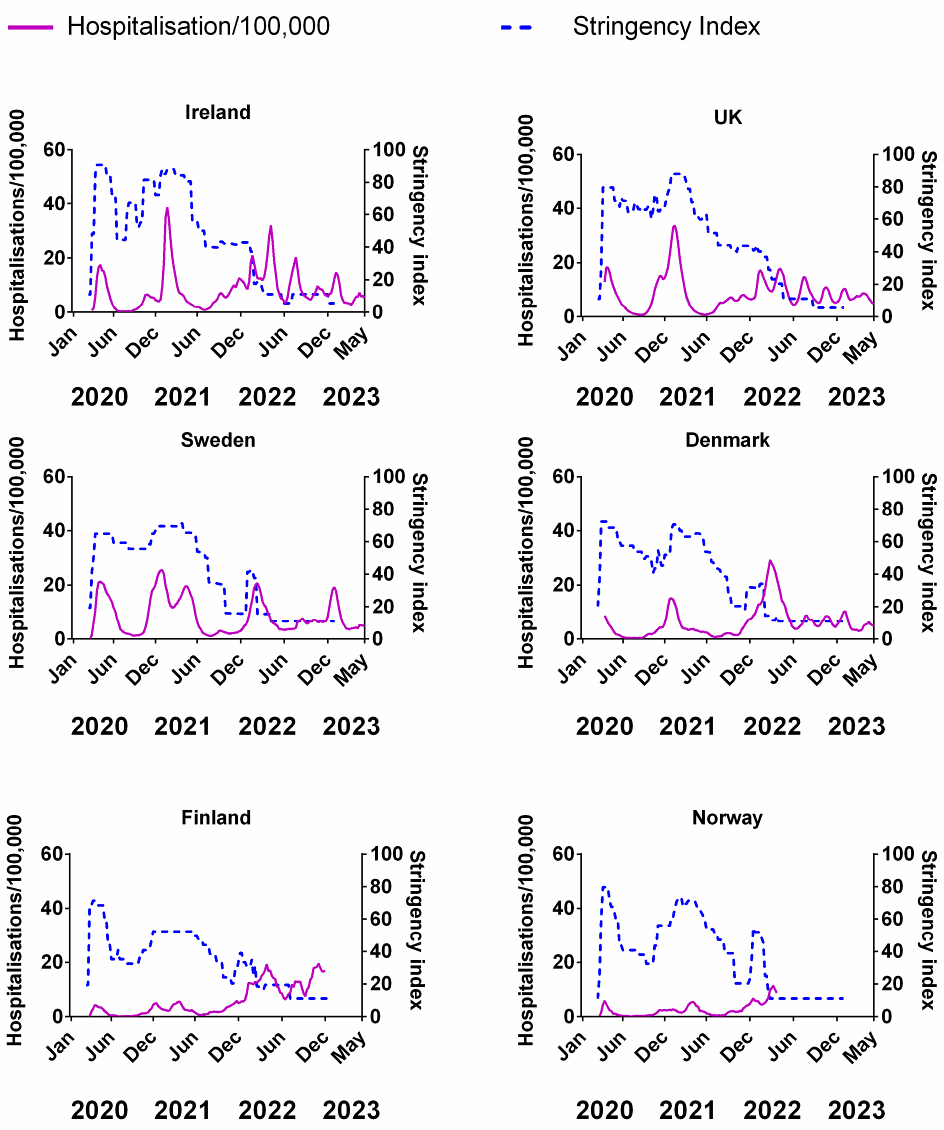

Figure S2c. Changes in NPI stringency compared to the progression of the COVID-19 pandemic in Northern Europe as measured by hospitalization. Data covering the period 1 March 2020 to 6 May 2023 was taken from "Our World in Data", https://ourworldindata.org/coronavirus; accessed 05/07/23 [55].

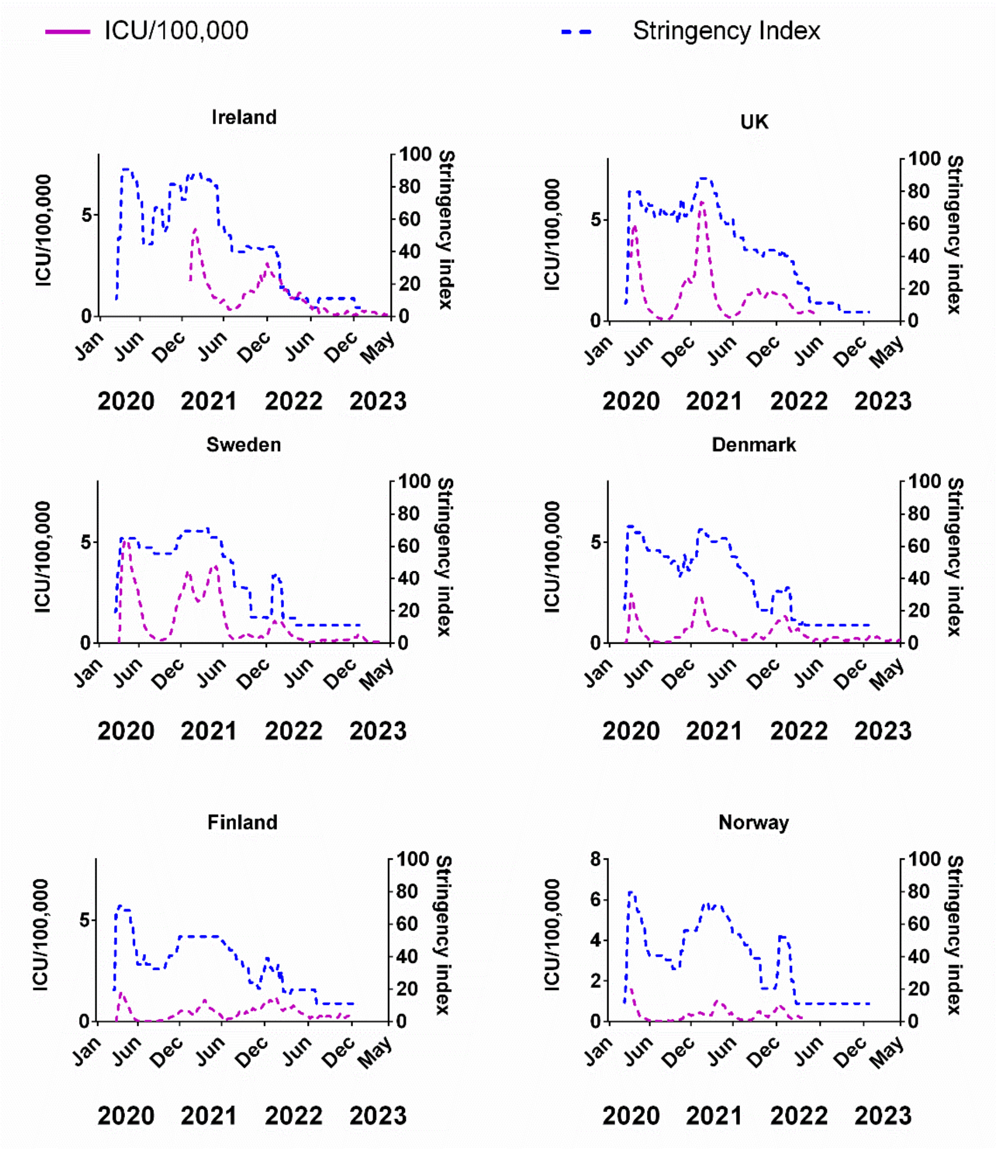

Figure S2d. Changes in NPI stringency compared to the progression of the COVID-19 pandemic in Northern Europe as measured by ICU occupancy. Data covering the period 1 March 2020 to 6 May 2023 was taken from “Our World in Data”, https://ourworldindata.org/coronavirus; accessed 05/07/23 [55].

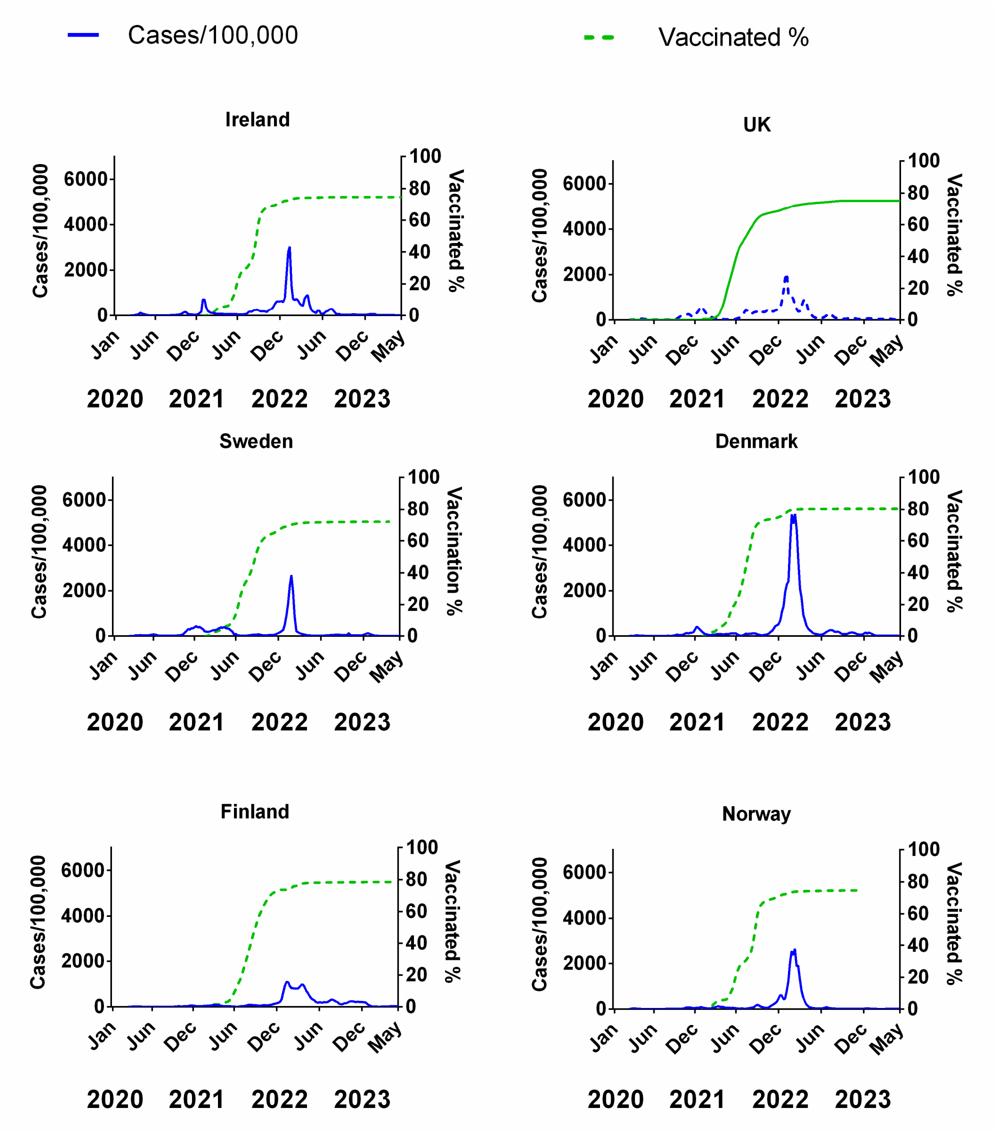

Figure S3a. Population wide vaccination programme compared to the progression of the COVID-19 pandemic in Northern Europe as measured by new cases. Data covering the period 1 March 2020 to 6 May 2023 was taken from "Our World in Data", https://ourworldindata.org/coronavirus; accessed 05/07/23 [55].

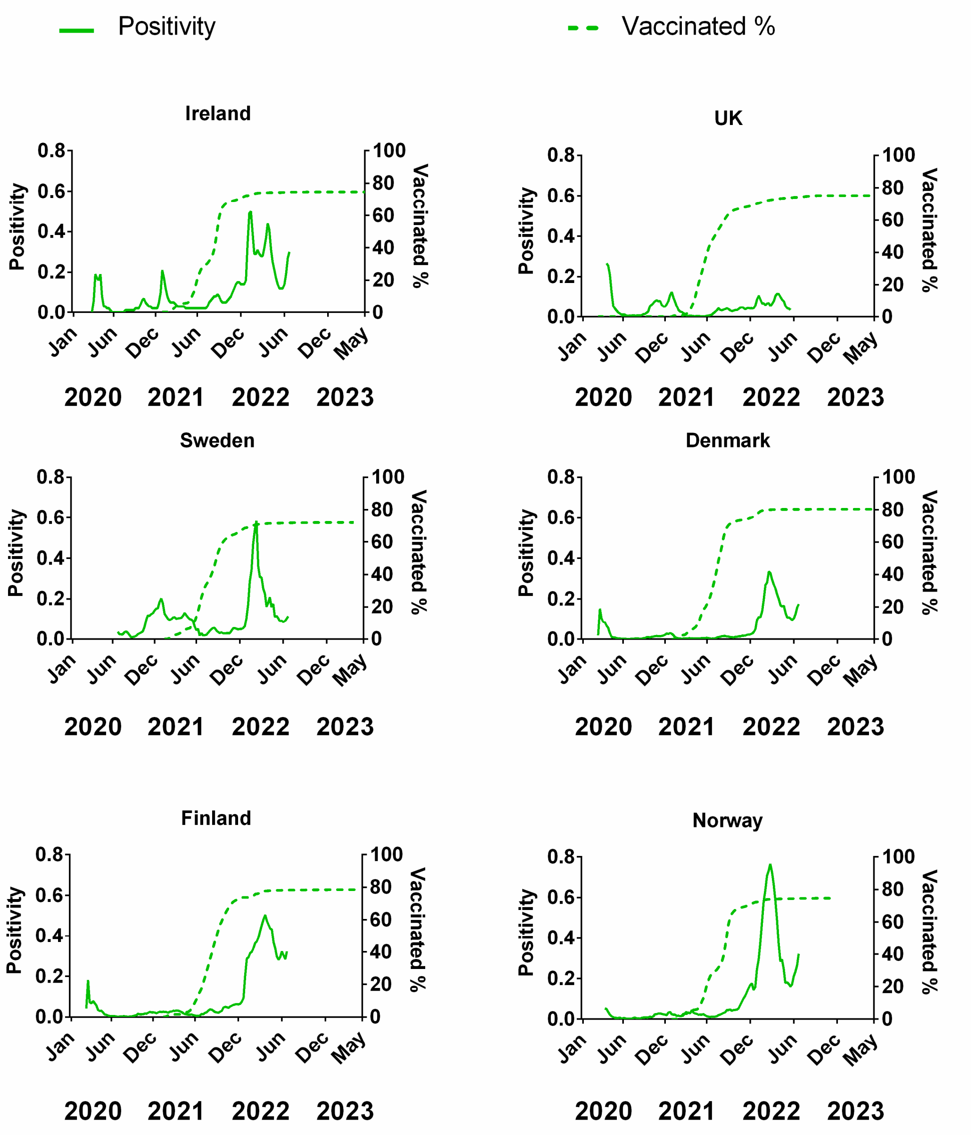

Figure S3b. Population wide vaccination programme compared to the progression of the COVID-19 pandemic in Northern Europe as measured by the positivity rate. Data covering the period 1 March 2020 to 6 May 2023 was taken from "Our World in Data", https://ourworldindata.org/coronavirus; accessed 05/07/23 [55].

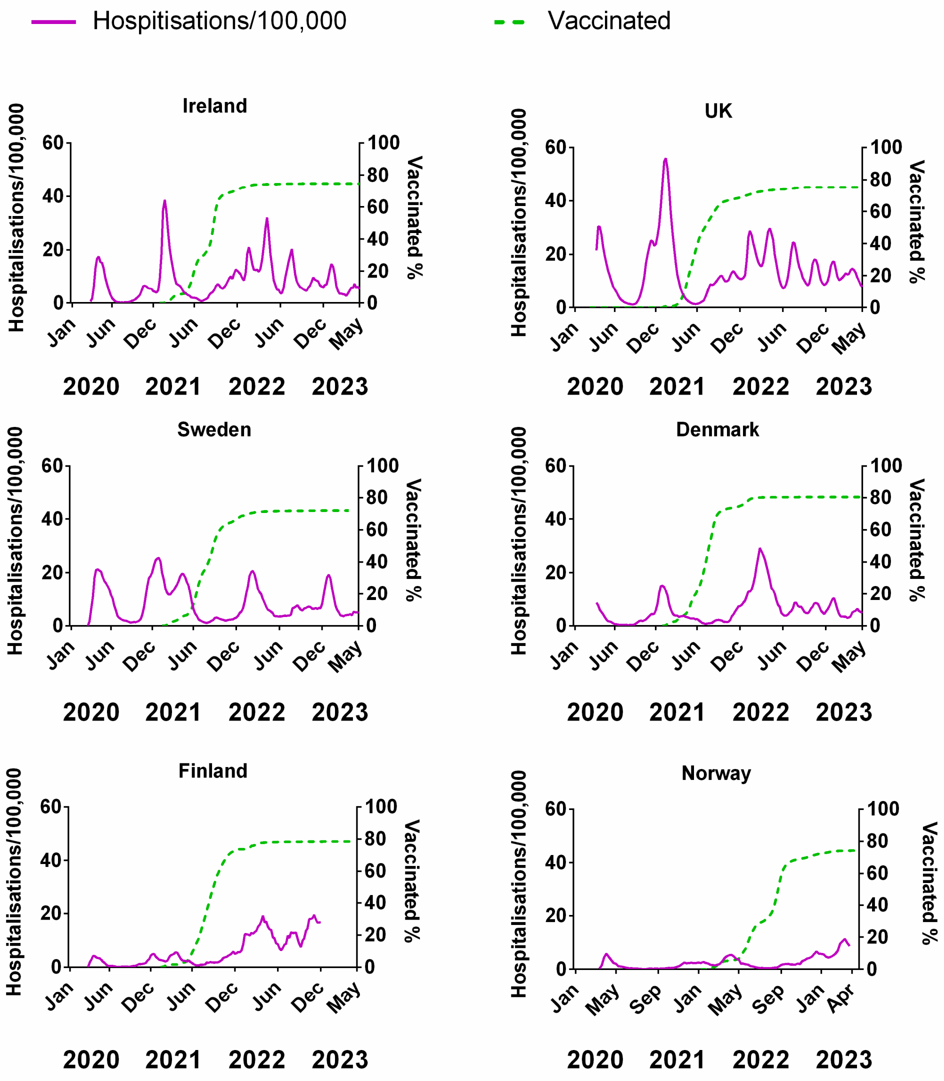

Figure S3c. Population wide vaccination programme compared to the progression of the COVID-19 pandemic in Northern Europe as measured by hospitalisation. Data cover-ing the period 1 March 2020 to 6 May 2023 was taken from "Our World in Data", https://ourworldindata.org/coronavirus; accessed 05/07/23 [55].

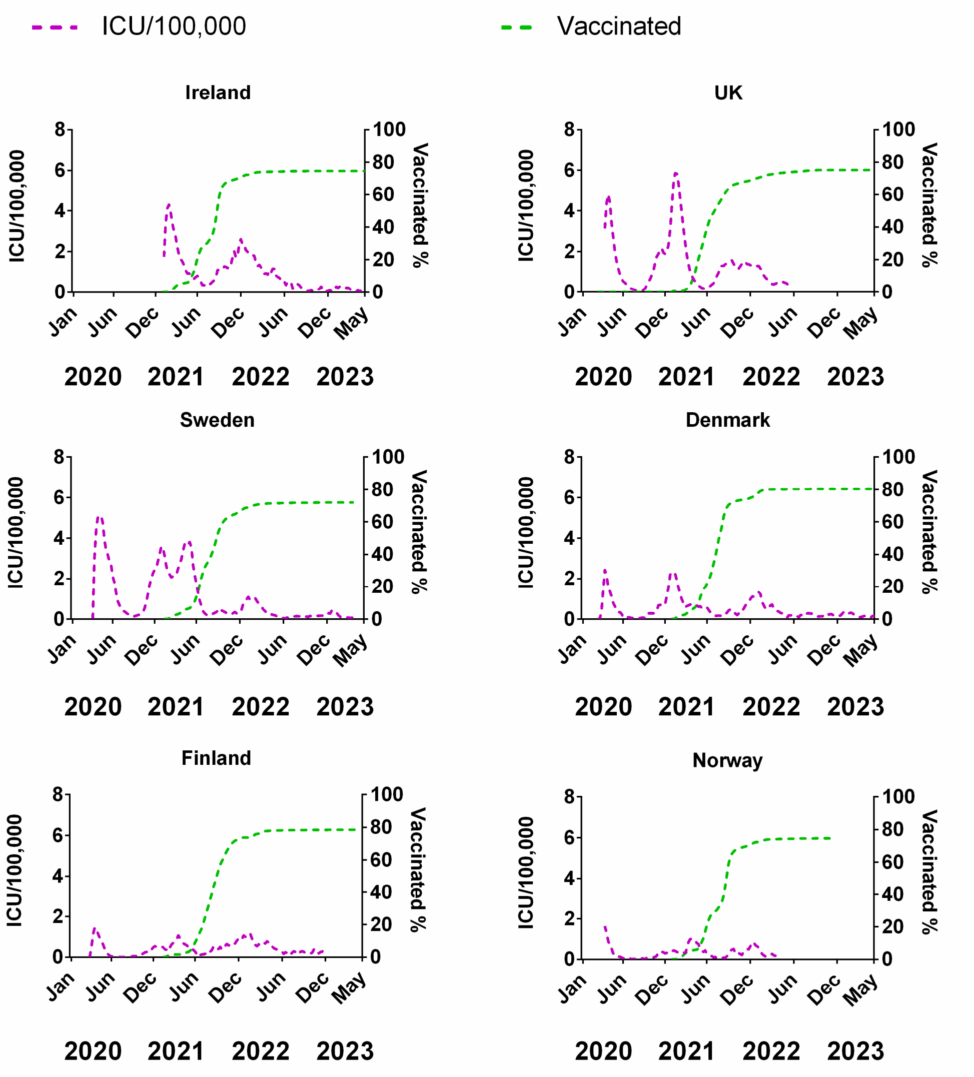

Figure S3d. Population wide vaccination programme compared to the progression of the COVID-19 pandemic in Northern Europe as measured by ICU occupancy. Data cover-ing the period 1 March 2020 to 6 May 2023 was taken from "Our World in Data", https://ourworldindata.org/coronavirus; accessed 05/07/23 [55].

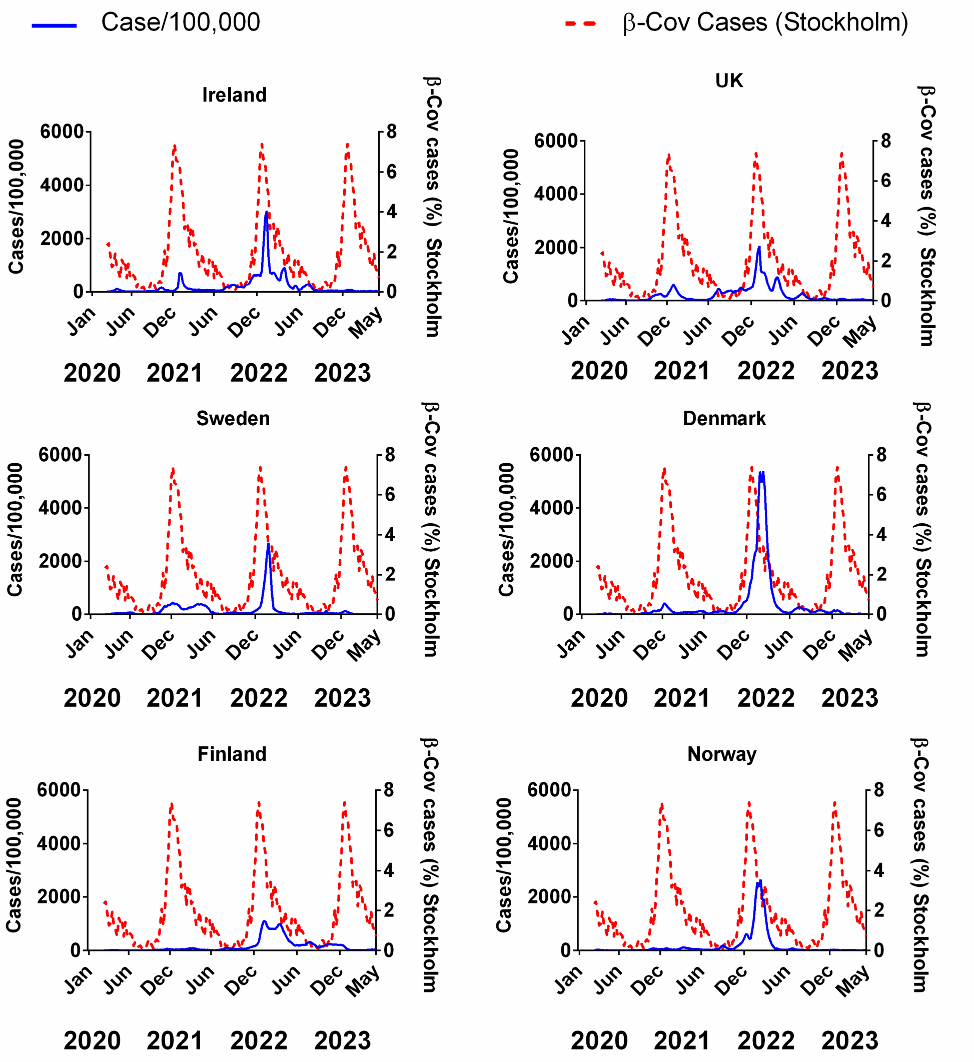

Figure S4a. Comparison of the average human beta-coronavirus cases in Stockholm over a ten year period with the progression of the COVID-19 pandemic in Northern Europe countries as measured by new cases. COVID-19 cases annotated per 100,000 of the population. Data covering the period 1 March 2020 to 6 May 2023 was taken from "Our World in Data", https://ourworldindata.org/coronavirus; accessed 05/07/23 [55]. and weekly beta-coronavirus (HCoV OC43 and HCoV HKU1) cases (1 January 2010 to 2 April 2020) from the University Hospital in Stockholm, Sweden [39].

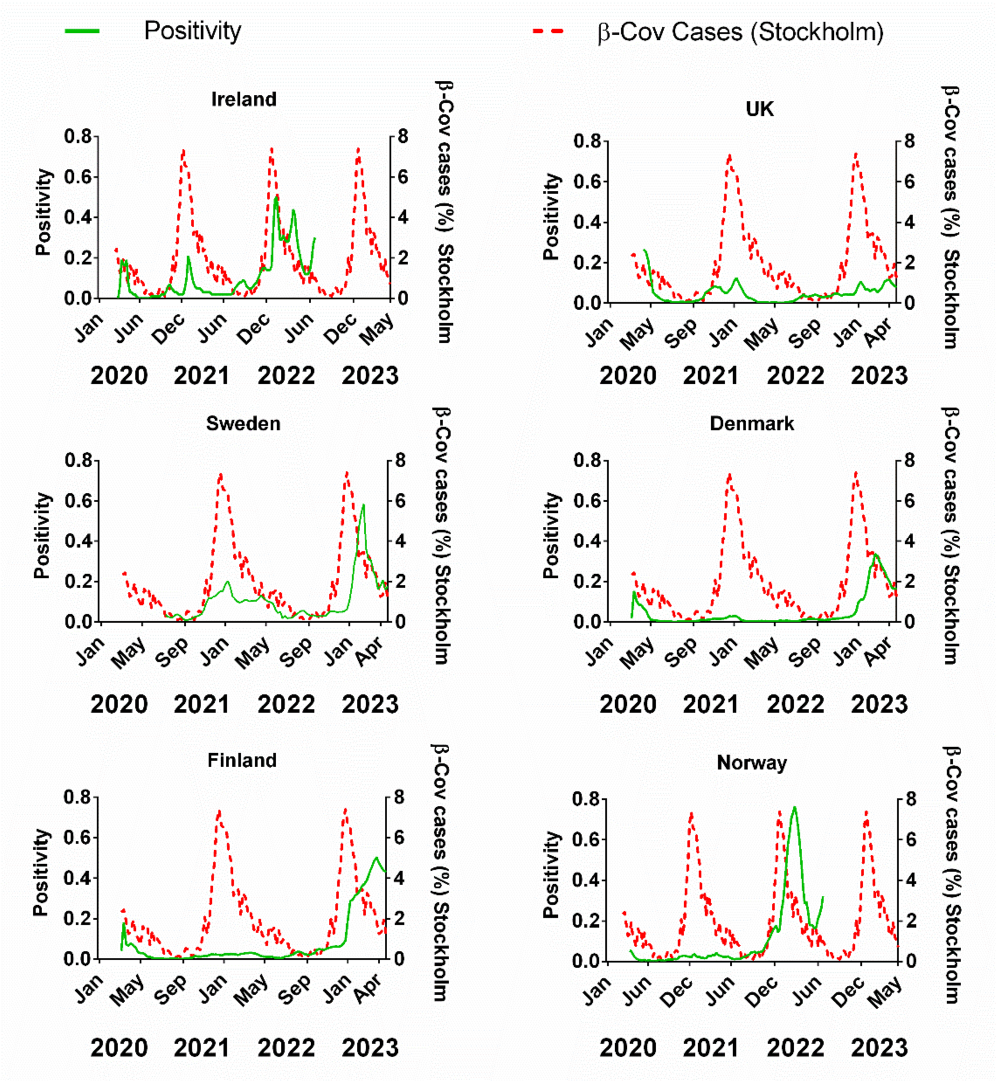

Figure S4b. Comparison of the average human beta-coronavirus cases in Stockholm over a ten year period with the progression of the COVID-19 pandemic in Northern Europe countries as measured by positivity rate. Data covering the period 1 March 2020 to 6 May 2023 was taken from "Our World in Data", https://ourworldindata.org/coronavirus; accessed 05/07/23 [55] and weekly beta-coronavirus (HCoV OC43 and HCoV HKU1) cases (1 January 2010 to 2 April 2020) from the University Hospital in Stockholm, Sweden [39].

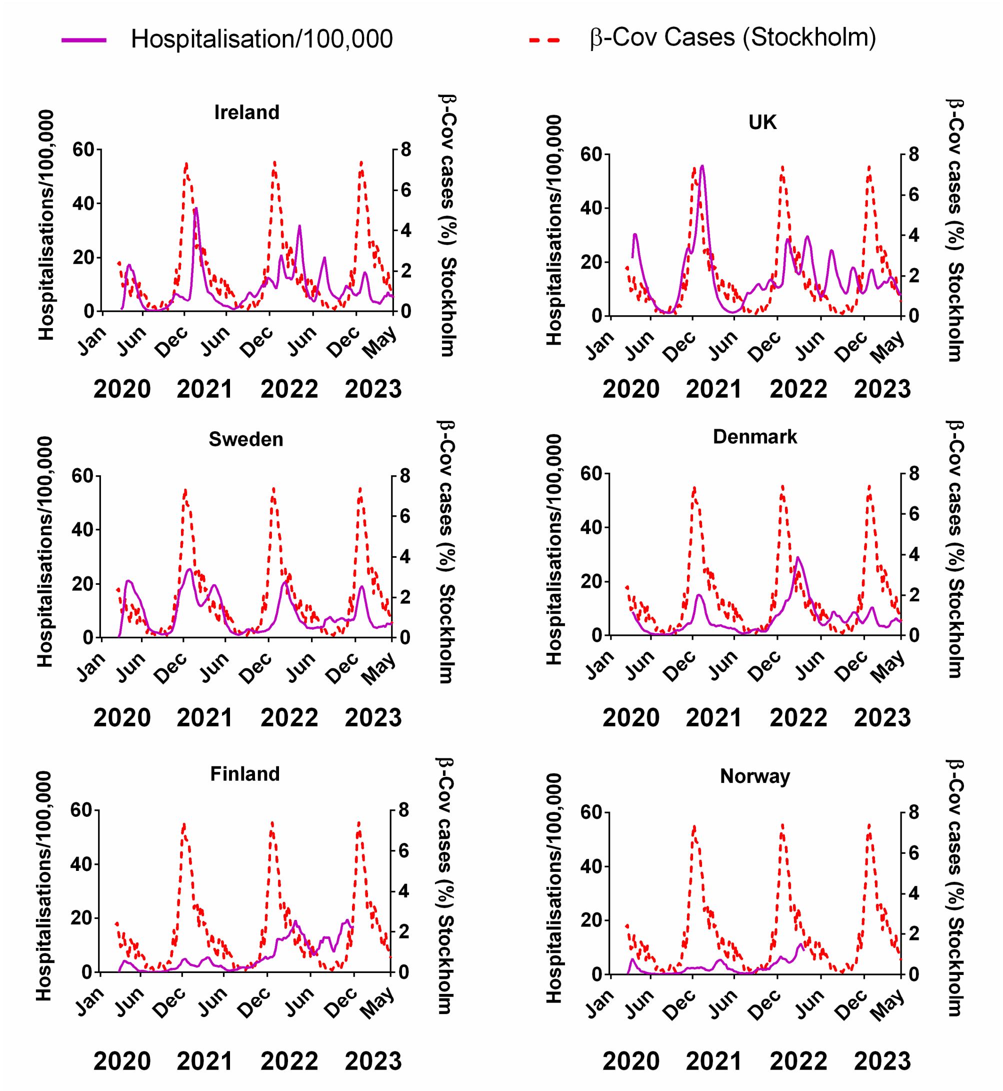

Figure S4c. Comparison of the average human beta-coronavirus cases in Stockholm over a ten year period with the progression of the COVID-19 pandemic in Northern Europe countries as measured by hospitalisation. Data covering the period 1 March 2020 to 6 May 2023 was taken from "Our World in Data", https://ourworldindata.org/coronavirus; accessed 05/07/23 [55] and weekly beta-coronavirus (HCoV -OC43 and HCoV -HKU1) cases (1 January 2010 to 2 April 2020) from the University Hospital in Stockholm, Sweden [39].

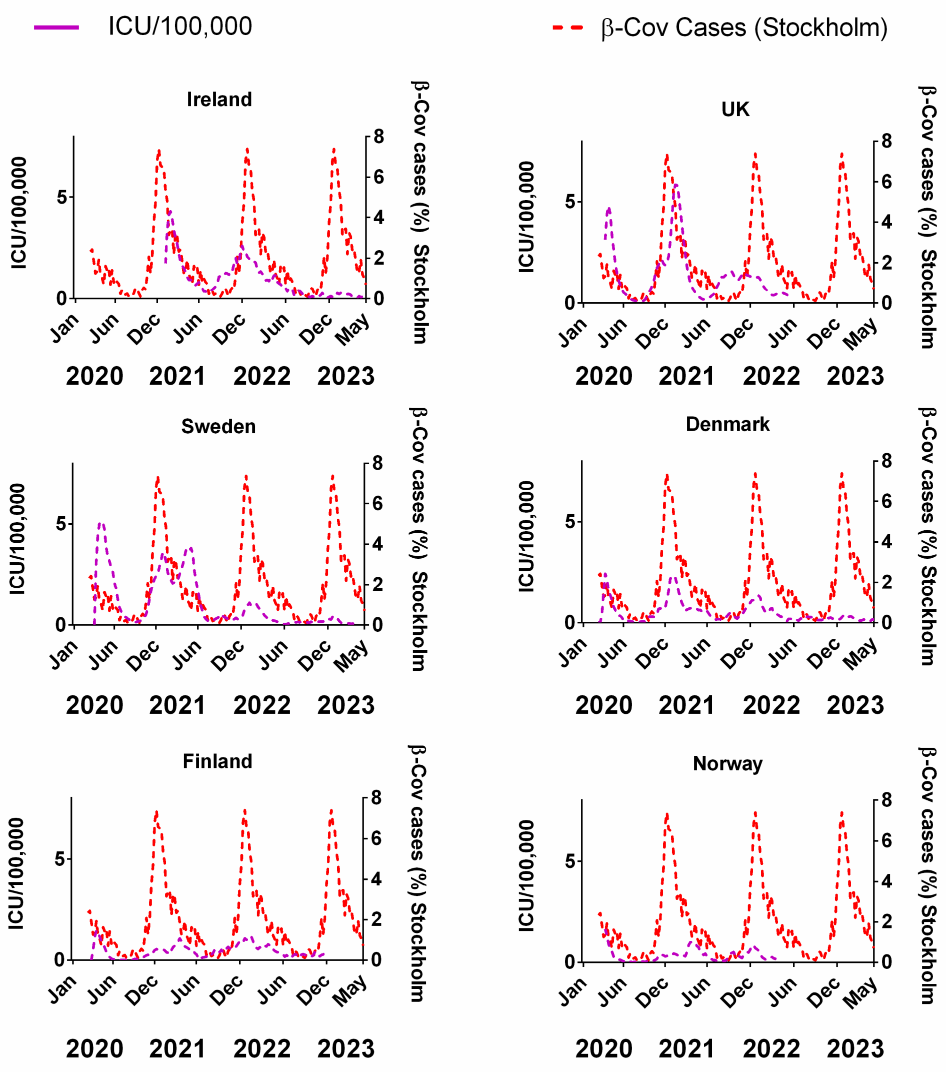

Figure S4d. Comparison of the average human beta-coronavirus cases in Stockholm over a ten year period with the progression of the COVID-19 pandemic in Northern Europe countries as measured by ICU occupancy. Data covering the period 1 March 2020 to 6 May 2023 was taken from "Our World in Data", https://ourworldindata.org/coronavirus; accessed 05/07/23 [55]. and weekly beta-coronavirus (HCoV -OC43 and HCoV -HKU1) cases (1 January 2010 to 2 April 2020) from the University Hospital in Stockholm, Sweden [39].

We also include the following data files in the supplementary information

Excel File S1. COVID-19 Epidemiological data for six Northern European countries. Epidemiological metrics used during COVID-19 pandemic and correlations of this data. Data from the period 1 March 2020 to 6 May 2023 was taken from "Our World in Data" https://ourworldindata.org/coronavirus; accessed 05/05/22. Average weekly beta-coronavirus (HCoV -OC43 and -HKU1) cases (1 January 2010 to 2 April 2020) from the University Hospital in Stockholm, Sweden.
